# Biocatalytic Production of a Nylon 6 Precursor from Caprolactone in Continuous Flow

**DOI:** 10.1002/cssc.202200811

**Published:** 2022-06-28

**Authors:** Maria Romero‐Fernandez, Christian M. Heckmann, Francesca Paradisi

**Affiliations:** ^1^ School of Chemistry University of Nottingham University Park NG7 2RD Nottingham United Kingdom; ^2^ Department of Chemistry Biochemistry and Pharmaceutical Sciences University of Bern Freiestrasse 3 3012 Bern Switzerland

**Keywords:** ϵ-caprolactone, biocatalysis, continuous flow, enzyme cascade, nylon 6

## Abstract

6‐Aminocaproic acid (6ACA) is a key building block and an attractive precursor of caprolactam, which is used to synthesize nylon 6, one of the most common polymers manufactured nowadays. (Bio)‐production of platform chemicals from renewable feedstocks is instrumental to tackle climate change and decrease fossil fuel dependence. Here, the cell‐free biosynthesis of 6ACA from 6‐hydroxycaproic acid was achieved using a co‐immobilized multienzyme system based on horse liver alcohol dehydrogenase, *Halomonas elongata* transaminase, and *Lactobacillus pentosus* NADH oxidase for in‐situ cofactor recycling, with >90 % molar conversion (m.c.) The integration of a step to synthesize hydroxy‐acid from lactone by immobilized *Candida antarctica* lipase B resulted in >80 % m.c. of ϵ‐caprolactone to 6ACA, >20 % of δ‐valerolactone to 5‐aminovaleric acid, and 30 % of γ‐butyrolactone to γ‐aminobutyric acid in one‐pot batch reactions. Two serial packed‐bed reactors were set up using these biocatalysts and applied to the continuous‐flow synthesis of 6ACA from ϵ‐caprolactone, achieving a space‐time yield of up to 3.31 g_6ACA_ h^−1^ L^−1^ with a segmented liquid/air flow for constant oxygen supply.

## Graphical abstract

**Packed tight**: In continuous cell‐free biocatalytic synthesis of 6‐aminocaproic acid from ϵ‐caprolactone by a packed‐bed reactor (PBR) system based on immobilized enzymes, *Candida antarctica* lipase B synthesizes hydroxy‐acid from lactone, and horse liver alcohol dehydrogenase and *Halomonas elongata* transaminase catalyze alcohol amination, with in‐situ cofactor recycling catalyzed by *Lactobacillus pentosus* NADH oxidase, achieving a space‐time yield of up to 3.31 g h^−1^ L^−1^.

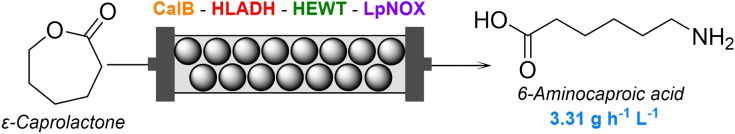

## Introduction

The replacement of petroleum‐derived chemicals with the ones derived from renewable feedstocks and/or bio‐based processes is instrumental to tackle global climate change and boost the transition from a fuel economy to a bioeconomy. In this transition, biocatalysis has a central role to play by providing more sustainable technological solutions to support industrial development.^[1]^ The largest fraction of all chemical products manufactured nowadays is represented by polymers and their precursors. In fact, the global polymer market was valued at 522.7 billion USD in 2017, and a 4 % annual increase is expected over 2019–2025. Despite these figures, almost all polymer building block chemicals are currently manufactured through petroleum‐based chemical processes, with their associated environmental costs. Therefore, there is an increasing global demand to replace petroleum‐based chemical processes with bio‐based chemical production from renewable resources within the manufacturing of polymer building blocks.^[2]^

Furthermore, nylon is one of the synthetic polymers with the largest annual production (nearly 7 million tons worldwide) and is used in a wide range of applications.^[3]^ As an alternative to the synthesis of nylon from petroleum‐derived chemicals, nylon can also be recycled from waste fishing nets, and this recycled polymer has been widely used as reinforcement in cementitious materials.^[4]^ Nylon 6, a homopolymer of 6‐aminocaproic acid (6ACA), and nylon 6,6, a copolymer of adipic acid and 1,6‐hexamethylenediamine, represent approximately 90 % of the nylon produced today.^[2]^ Nylon 6 is currently synthesized from caprolactam via ring‐opening polymerization.^[5]^ In addition, caprolactam is mainly synthesized from cyclohexanone oxime, although efforts are directed towards its synthesis from sustainable bio‐resources. 6ACA is an attractive precursor for caprolactam and can be obtained from renewable resources.^[6]^ The whole‐cell based biosynthesis of 6ACA has been reported, for example, using engineered *Escherichia coli* cells, yielding up to 160 mg L^−1^ after 120 h of batch fermentation and up to 2 g L^−1^ after 72 h of fed‐batch fermentation.[^3^, ^7^] Moreover, the biocatalytic conversion of adipic acid to 6ACA was developed by the use of purified carboxylic acid reductase and transaminase (TA) and supplemented with cofactor regenerating systems for ATP, NADPH, and amine donor, achieving 95 % conversion at 10 mm scale.^[2]^

In addition to 6ACA, 5‐aminovaleric acid (5AVA), has drawn much attention in industry and academia for its potential application in nylon synthesis. The biosynthesis of 5AVA has also been reported, reaching up to 39.93 g L^−1^ with a fed‐batch fermentation process using an engineered strain of *Corynebacterium glutamicum*.^[8]^ γ‐Aminobutyric acid (GABA) can be also used for the synthesis of nylon 4. This four‐carbon amino acid is the main inhibitory neurotransmitter in human cortex. It also has a potential use as food additive or dietary supplement due to its physiological functions, like blood pressure decrease, anxiety, and diabetes inhibition. Different biosynthetic pathways, based on microbial fermentations, have been described for the production of GABA, yielding up to 26.32 g L^−1^ GABA in *C. glutamicum* with a fed‐batch fermentative process.^[9]^

As an alternative to whole‐cell biocatalysis, which is often associated with problems of cross‐reactivity with cellular metabolites, decomposition of substrates and products through competing cellular reactions,^[10]^ and potential substrate diffusion limitations into cells, the use of isolated enzyme biocatalysis has been implemented for a wide range of chemical reactions.^[11]^ Nevertheless, process costs derived from enzymes separation from the product‐rich stream and limited operational stability of enzymes mainly hinders the application of isolated enzyme biocatalysis in industrial settings, and the use of immobilized enzyme biocatalysts has emerged as a prominent approach to overcome these drawbacks.^[10]^ Enzyme immobilization provides the additional benefit of enabling the setup of packed‐bed reactors (PBRs) for continuous flow biocatalyzed reactions,^[12]^ meeting the increasing popularity of flow technologies in both academia and industry.^[13]^ The use of continuous‐flow PBRs based on immobilized biocatalysts not only minimizes biocatalysts exposure to shear forces, but also it overcomes the limited particle loading in stirred tank reactors. The synergy of an efficient immobilization strategy of (multi)enzymatic systems, reactor engineering and control, and process integration for in situ product removal and cofactor recycling can contribute to biocatalytic processes intensification.[^14^, ^15^]

On the other hand, lactones, internal cyclic monoesters, are ubiquitous in nature and have been identified in all major classes of foods, including fruits, vegetables, nuts, meat, milk products, and baked products, contributing to taste and flavor. The organoleptically important lactones generally have γ‐ or δ‐lactone structures (five‐ or six‐membered ring structures), while a few are macrocyclic.^[16]^ The largest volume lactone is caprolactone, which is primarily produced from cyclohexanone using Baeyer‐Villiger (BV) oxidation chemistry with peracetic acid at 50 °C with 85–90 % selectivity.^[1]^ The utilization of lignin as a feedstock for the production of bio‐based chemicals is receiving increasing attention.[^17^, ^18^] The synthesis of caprolactone from monomers derived from lignin depolymerization has also been described, through the oxidation of cyclohexanols, which are produced from the hydrodeoxygenation of lignin depolymerization products, to the corresponding cyclohexanone.^[17]^ Furthermore, γ‐valerolactone and δ‐valerolactone can be obtained from nonedible lignocellulose biomass, through catalytic coupling of 1,5‐pentanediol dehydrogenation and ethyl levulinate hydrogenation.^[19]^ γ‐Valerolactone can be also synthesized by the hydrogenation of levulinic acid, which is produced from acid hydrolysis of lignocellulosic materials.^[20]^ Likewise, γ‐butyrolactone can be produced from hydrogenative lactonization of the renewable succinic acid obtained from the microbial fermentation of sugar.^[21]^ Therefore, designing an alternative synthetic route of 6ACA and other potential nylon precursors from the corresponding lactones is relevant given their ubiquity in nature and the developed strategies to obtain lactones from renewable lignocellulosic biomass. In a first example of this kind, López‐Gallego and co‐workers reported on a one‐pot cell‐free biocatalytic cascade synthesis in batch of short‐chain ω‐hydroxyacids from diols where five enzymes are combined. The lactones, as key intermediates, are produced in the second step of the cascade and hydrolyzed to the diols by a lactonase.^[22]^ Within the same ambitious EU project (H2020 ERACoBioTech, HOMBIOCAT), we are now targeting the production of 6ACA, 5AVA, and GABA from the corresponding lactones (as they are more easily accessible than the diols) that can be eventually accessed through the ambitious combination of both systems.

**Scheme 1:**
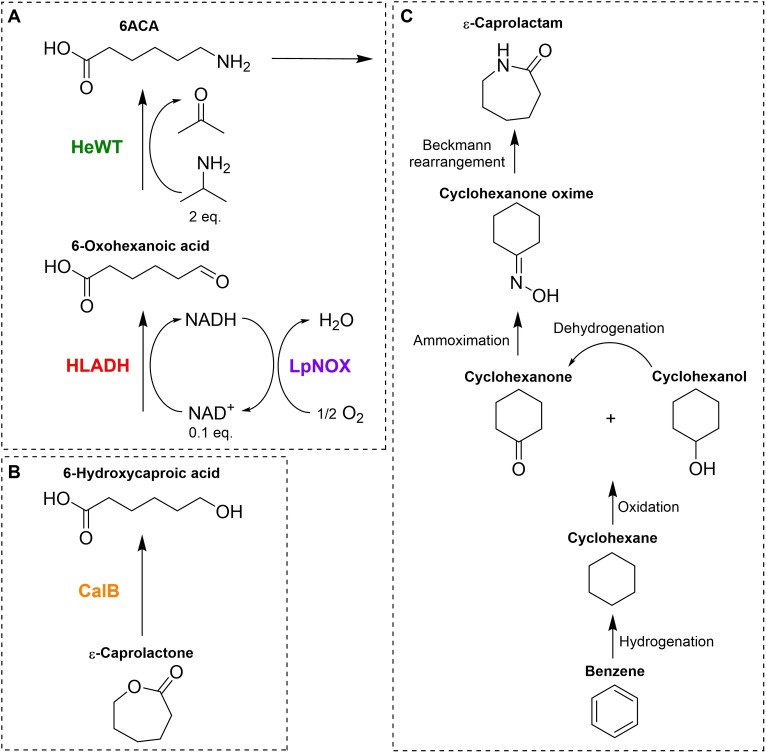
Multienzyme system catalyzing the cell‐free biosynthesis of 6ACA, an attractive precursor of ϵ‐caprolactam, from ϵ‐caprolactone by a two‐step process: (A) functional group interconversion of the alcohol group of 6‐hydroxycaproic acid to the corresponding primary amine to produce 6ACA in one‐pot and (B) hydrolysis of ϵ‐caprolactone to obtain 6‐hydroxycaproic acid. (C) Most common route of chemical synthesis of ϵ‐caprolactam from petroleum‐derived chemicals.

Therefore, in this work, we describe the synthesis of 6ACA, 5AVA, and GABA based on immobilized enzyme biocatalysts, and the production in flow of 6ACA from ϵ‐caprolactone using commercial *Candida antarctica* lipase B (CalB) for the first hydrolytic step. Overall, this biosynthetic strategy is based on a two‐step process: the enzymatic hydrolysis of lactones to obtain hydroxy‐acid intermediates, and the alcohol amination of the latter to form ω‐amino acids. For this purpose, a previously developed co‐immobilization strategy^[23]^ has been exploited to achieve a high‐yielding system to catalyze the one‐pot enzymatic amination of the alcohol group of 6‐hydroxycaproic acid, with in situ cofactor recycling, and optimized for the synthesis of 6ACA (Scheme 1). This system has been combined with a biocatalyzed step of hydrolysis of the different lactones to yield the corresponding hydroxy‐acid intermediates and develop the one‐pot synthesis of 6ACA, 5AVA, and GABA from lactones. Additionally, these two systems were integrated into two serial PBRs and used for the continuous‐flow synthesis of 6ACA from ϵ‐caprolactone, which can be produced from lignin, thus contributing to the replacement of petroleum‐derived feedstocks in the manufacturing of nylon 6.

## Results and Discussion

### One‐pot multienzyme system to synthesize 6ACA from 6‐hydroxycaproic acid

Focusing initially on the conversion of the hydroxy acids to the amino acids, a one‐pot multienzyme system to catalyze the synthesis of the primary amine of 6ACA from the corresponding alcohol of 6‐hydroxycaproic acid was designed by a two‐step process: the first alcohol oxidation to form a carbonyl group, and the second amination of the later to form the primary amine, as previously reported.[^23^, ^24^, ^25^, ^26^, ^27^, ^28^]

Initial attempts at using alcohol oxidases (AOX) to produce the aldehyde intermediate in Scheme 1 were unsuccessful. AOXs from *Candida tropicalis*,^[29]^ and from *Pleurotus eryngii*,^[30]^ expressed insolubly in *E. coli*. And in reactions with the commercially sourced AOX from *Pichia pastoris* and *Candida boidinii*, and glucose oxidase from *Aspergilus niger* [tested in combination with *Halomonas elongata* transaminase (HeWT)^[31]^] <20 % of conversion to the final product were observed (Figure S1). Finally, a previously developed multienzyme system was tested to catalyze the synthesis of 6ACA from 6‐hydroxycaproic acid: horse liver alcohol dehydrogenase (HLADH),[^32^, ^33^] HeWT, and *Lactobacillus pentosus* NADH oxidase (LpNOX)[^34^, ^35^] for in situ nicotinamide adenine dinucleotide (NAD^+^) cofactor regeneration (Scheme 1A).^[23]^ The catalytic activity of this multienzyme system on the alcohol group of 6‐hydroxycaproic acid was tested in batch reactions at a 10 mm scale with 0.1 mol equivalents NAD^+^, using isopropylamine (IPA) as the amino‐donor (2 mol equivalents), 0.1 mm pyridoxal 5’‐phosphate (PLP) and 1 mm flavin adenine dinucleotide (FAD). High molar conversion (m.c.) of 96 % to 6ACA was achieved after 48 h under the specified reaction conditions (Table 1). At 50 and 100 mm scales, respectively, 59 and 29 % m.c. were obtained after 48 h of reaction under the same conditions (Table 1). Hence, the multienzyme system consisting of HLADH, HeWT, and LpNOX can catalyze the functional group interconversion reaction of the alcohol of 6‐hydroxycaproic acid to the corresponding primary amine to synthesize 6ACA, with in‐situ regeneration of NAD^+^. This system was further explored.

**Table 1:** Synthesis of 6ACA in batch reactions catalyzed by soluble HLADH (1.5 mg mL^−1^), HEWT (0.75 mg mL^−1^), and LpNOX (0.25 mg mL^−1^).

Scale [mm]	m.c.^[a]^ [%]			STY^[b]^ [g_6ACA_ L^−1^ h^−1^]	
	2 h	24 h	48 h	2 h	24 h
10	65	107	96	0.42	0.06
50	19	51	59	0.64	0.14
100	4	23	29	0.25	0.12

[a] Determined by HPLC by following product formation using a calibration curve. [b] Space‐time yield calculated as described in the Supporting Information. Reaction conditions: 10, 50, or 100 mm 6‐hydroxycaproic acid in 50 mm potassium phosphate buffer pH 8, 0.1 equiv. NAD^+^ (1, 5, or 10 mm), 2 equiv. IPA (20, 100, or 200 mm), 1 mm FAD, and 0.1 mm PLP. *T*=30 °C. Reaction volume=1 mL. Mean values of triplicate reactions.

### One‐pot synthesis of 6ACA from 6‐hydroxycaproic acid by a co‐immobilized enzyme biocatalyst

To increase the operational stability of this multienzyme system, and therefore improve the reaction conversion, this multienzyme system was immobilized on a methacrylate‐based porous beads carrier. For that purpose, a previously developed strategy of co‐immobilization of this multienzyme system based on tailored immobilization chemistries for each enzyme was followed.^[23]^ The resulting co‐immobilized multienzyme system (Table S1) was used to catalyze the synthesis of 6ACA from 6‐hydroxycaproic acid in batch reactions under the same conditions as used with the soluble enzymes. Significantly higher m.c. was obtained after 24 h of reaction at 50 (91 %) and 100 mm (59 %) with the co‐immobilized multienzyme system compared to its soluble counterpart (Table 2), thus highlighting the improved overall biocatalyst operational stability obtained upon enzyme immobilization, as previously observed for this multienzyme system.^[23]^

**Table 2:** Synthesis of 6ACA in batch reaction catalyzed by 0.1 g of the co‐immobilized multienzyme system consisting of HLADH, HEWT, and LpNOX per mL of reaction.

Scale [mm]	m.c.^[a]^ [%]			STY^[b]^ [g_6ACA_ L^−1^ h^−1^]	
	2 h	24 h	48 h	2 h	24 h
10	55	85	75	0.36	0.05
50	17	91	83	0.55	0.25
100	7	59	60	0.47	0.32

[a] Determined by HPLC by following product formation using a calibration curve. [b] Space‐time yield calculated as described in the Supporting Information. Reaction conditions: 10, 50, or 100 mm 6‐hydroxycaproic acid in 50 mm potassium phosphate buffer pH 8, 0.1 equiv. NAD^+^ (1, 5, or 10 mm), 2 equiv. IPA (20, 100, or 200 mm), 1 mm FAD, and 0.1 mm PLP. *T*=30 °C. Reaction volume=1 mL. Mean values of triplicate reactions.

The in‐house purified HLADH was then replaced by a commercially sourced HLADH, thus avoiding the step of enzyme expression and purification to obtain HLADH biocatalyst. A commercial preparation containing HLADH, as well as other proteins resulting from HLADH expression in *E. coli*, was immobilized by the multipoint‐covalent attachment methodology on glyoxyl groups.^[36]^ HEWT and LpNOX enzymes were respectively immobilized by the amino‐epoxy strategy and by ionic adsorption, following the sequential approach previously reported (Table S2).^[23]^ The ratio of commercial HLADH to HeWT loadings (Figure S2), and the individual loading of commercial HLADH into the carrier (Figures S3 and S4), were optimized for the reaction of synthesis of 6ACA from 6‐hydroxycaproic acid in batch at a 10 mm scale. The resulting optimized immobilized multienzyme system based on the commercially sourced HLADH was used to catalyze the batch reaction of synthesis of 6ACA from 6‐hydroxycaproic acid at 50 and 100 mm scales (Table 3). After 48 h of reaction under the same conditions, 82 and 71 % m.c. were attained at 50 and 100 mm scales, respectively, which were very similar to the ones obtained with the immobilized multienzyme system based on purified HLADH (Table 2). The decrease of catalyst productivity over time could be explained by the overall limited operational stability of the resulting co‐immobilized biocatalyst due to the relatively low stability of immobilized LpNOX, as previously described.^[23]^

**Table 3:** Synthesis of 6ACA in batch reaction catalyzed by 0.1 g of the co‐immobilized multienzyme system consisting of commercial HLADH (loading 40 mg g_carrier_^−1^), HEWT (loading 6 mg g_carrier_^−1^), and LpNOX (loading 5 mg g_carrier_^−1^) per mL of reaction.

Scale [mm]	m.c.^[a]^ [%]			Catalyst productivity^[b]^ [μmol_6ACA_ h^−1^ mg_enzyme_^−1^]			STY^[c]^ [g_6ACA_ L^−1^ h^−1^]	
	2 h	24 h	48 h	2 h	24 h	48 h	2 h	24 h
50	23	83	82	1.10	0.34	0.17	0.74	0.23
100	15	69	71	1.48	0.57	0.29	0.99	0.38

[a] Determined by HPLC by following product formation using a calibration curve. [b] Catalyst productivity calculated as described in the Supporting Information. [c] Space‐time yield calculated as described in the Supporting Information. Reaction conditions: 50 or 100 mm 6‐hydroxycaproic acid in 50 mM potassium phosphate buffer pH 8, 0.1 equiv. NAD^+^ (5 or 10 mm), 2 equiv. IPA (100 or 200 mm), 1 mm FAD, and 0.1 mm PLP. *T*=30 °C. Reaction volume=1 mL. Mean values of triplicate reactions.

### Hydrolysis of lactones by immobilized enzyme in batch

Next, the hydrolysis reaction of ϵ‐caprolactone to give 6‐hydroxycaproic acid was explored using Novozym® 435, a commercially available immobilized preparation of *Candida antarctica* lipase B (CalB) (Scheme 1b). >90 % m.c. to 6‐hydroxycaproic acid was obtained in batch reaction at a 100 mm scale after 2 h (Table 4). This immobilized CalB biocatalyst was also used to catalyze the reaction of synthesis of 5‐hydroxypentanoic acid and 4‐hydroxybutanoic acid from δ‐valerolactone and γ‐butyrolactone, respectively, under the same reaction conditions as used for 6‐hydroxycaproic acid. >90 % m.c. to 5‐hydroxypentanoic acid was attained, but only 46 % m.c. to γ‐butyrolactone was achieved within around 2 h of reaction (Table 4). Therefore, the immobilized CalB biocatalyst can catalyze the reaction of hydrolysis of ϵ‐caprolactone and δ‐valerolactone to, respectively, yield 6‐hydroxycaproic acid and 5‐hydroxypentanoic acid, although it showed limited activity towards the hydrolysis of γ‐butyrolactone to form 4‐hydroxybutanoic acid.

**Table 4:** Synthesis of 6‐hydroxycaproic acid, 5‐hydroxypentanoic acid, and 4‐hydroxybutanoic acid in batch reactions catalyzed by 25 mg mL^−1^ of immobilized CalB (Novozym® 435).

Substrate	m.c.^[a]^ [%]	STY^[b]^ [g L^−1^ h^−1^]
ϵ‐caprolactone^[c]^	99	6.5
δ‐valerolactone^[d]^	93	7.3
γ‐butyrolactone^[e]^	46	2.7

[a] Determined by ^1^H NMR spectroscopy, using relative integrations of starting material and product.^[37]^ [b] Space‐time yield calculated as described in the Supporting Information. Reaction conditions: 100 mm ϵ‐caprolactone, δ‐valerolactone, or γ‐butyrolactone, in 100 mm potassium phosphate buffer pH 8. *T*=37 °C. Reaction volume=0.4 mL. [c] At 2 h of reaction. [d] At 1.5 h of reaction. [e] At 1.75 h of reaction.

### Continuous‐flow hydrolysis of lactones

A continuous‐flow PBR was set up using the immobilized CalB biocatalyst (CalB PBR) and applied to the hydrolysis reactions of ϵ‐caprolactone, δ‐valerolactone, and γ‐butyrolactone to, respectively, synthesize 6‐hydroxycaproic acid, 5‐hydroxypentanoic acid, and 4‐hydroxybutanoic acid (Table 5). A liquid phase containing 100 mm substrate in 100 mm potassium phosphate buffer was flowed into CalB PBR, at 37 °C, with a residence time of 10 min, obtaining 94 % m.c. to 6‐hydroxycaproic acid, 85 % m.c. to 5‐hydroxypentanoic acid, and 36 % to 4‐hydroxybutanoic acid. In case of 5‐hydroxypentanoic acid, increasing residence time to 15 min pushed the m.c. to 92 %. However, for 4‐hydroxybutanoic acid increasing the residence time to 20 min only resulted in a slight increase in m.c. to 47 %. With a residence time of 20 min and a temperature of 45 °C, the m.c. to 4‐hydroxybutanoic acid was 50 %, thus highlighting the limited applicability of CalB towards the hydrolysis reaction of γ‐butyrolactone, as was observed in batch (Table 4).

**Table 5:** Continuous biocatalytic production of 6‐hydroxycaproic acid, 5‐hydroxypentanoic acid, and 4‐hydroxybutanoic acid in a PBR.

Substrate	T [°C]	Residence time [min]	m.c.^[a]^ [%]	STY^[b]^ [g_6ACA_ L^−1^ h^−1^]
ϵ‐caprolactone	37	10	94	74
δ‐valerolactone	37	10	85	60
	37	15	92	44
γ‐butyrolactone	37	10	36	22
	37	20	47	15
	45	20	50	16

[a] Determined by ^1^H NMR spectroscopy, using relative integrations of starting material and product.^[37]^ [b] Space‐time yield calculated as described in the Supporting Information. Substrate solution: 100 mm ϵ‐caprolactone, δ‐valerolactone, or γ‐butyrolactone in 50 mm potassium phosphate buffer pH 8, 4 % DMSO. *T*=37–45 °C. *P*=atmospheric pressure. Reactor volume=2.5 mL. Residence time=10–20 min.

### One‐pot synthesis of 6ACA, 5AVA, and GABA from lactones by immobilized enzyme biocatalysts in batch

The one‐pot biosynthesis of 6ACA from ϵ‐caprolactone was carried out by combining the immobilized CalB biocatalyst and the co‐immobilized multienzyme system consisting of commercial HLADH, HeWT, and LpNOX, in batch reactions (Scheme 1). At a 50 mm scale, using 0.1 equiv. of NAD^+^, 2 equiv. of IPA, 0.1 mm PLP, and 1 mm FAD, only 9 % m.c. to 6ACA was obtained after 48 h of reaction (Table 6). Using the same conditions to synthesize 5AVA and GABA from δ‐valerolactone and γ‐butyrolactone, m.c. values of 1 and 14 % to 5AVA and GABA were obtained, respectively (Table 5). The low m.c. values are due to a drop in pH (to ≈5.5 under these reaction conditions) resulting from the lactone ring opening.

**Table 6:** Synthesis of 6ACA, 5AVA, and GABA in batch reaction catalyzed by 0.01 g of immobilized CalB (Novozym® 435) and 0.1 g of the co‐immobilized multienzyme system consisting of commercial HLADH, HEWT, and LpNOX per mL of reaction.

Phosphate buffer [mm]	Substrate	m.c.^[a]^ [%]		STY^[b]^ [g L^−1^ h^−1^]
		24 h	48 h	24 h
50	ϵ‐caprolactone	8	9	0.002
	δ‐valerolactone	1	1	0.00
	γ‐butyrolactone	11	14	0.02
200	ϵ‐caprolactone	84	81	0.46
	δ‐valerolactone	17	24	0.08
	γ‐butyrolactone	21	30	0.09

[a] Determined by HPLC by following product formation using a calibration curve. [b] Space‐time yield calculated as described in the Supporting Information. Reaction conditions: 50 mm ϵ‐caprolactone, δ‐valerolactone, or γ‐butyrolactone, in 50 or 200 mm phosphate buffer pH 8, 0.1 equiv. NAD^+^ (5 mm), 2 equiv. IPA (100 mm), 1 mm FAD, and 0.1 mm PLP. *T*=30 °C. Reaction volume=1 mL. Mean values of triplicate reactions.

To compensate the pH drop, an out‐buffering strategy was implemented under otherwise identical conditions. With this approach, 81 % m.c. to 6ACA was obtained. Conversions to 5AVA and GABA also improved, reaching 24 and 30 % m.c., respectively (Table 6). The obtained value of space‐time yield (STY) for 6ACA, 0.46 g_6ACA_ L^−1^ h^−1^, is one order of magnitude higher than the value calculated from reported data on the biosynthesis of 6ACA using engineered *E. coli* cells, which is estimated to be approximately 0.03 g_6ACA_ L^−1^ h^−1^ (at 72 h of fed‐batch fermentation).[^3^, ^7^] Remarkably, this represents the first example of synthesis of 6ACA from the corresponding lactone by means of cell‐free biocatalysis, which puts a step forward in the sustainability of the nylon 6 polymer manufacturing, with significant potential in transitioning to lignin‐derived feedstocks to provide the ϵ‐caprolactone substrate.^[17]^

### Continuous‐flow synthesis of 6ACA from ϵ‐caprolactone

**Scheme 2:**
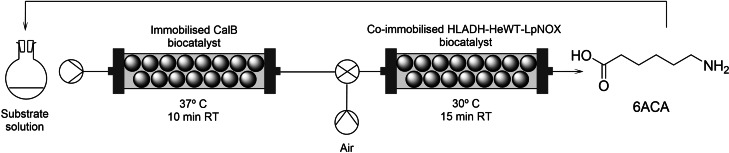
Continuous biocatalytic production of 6ACA from ϵ‐caprolactone in two serial PBRs implementing segmented liquid‐gas flow composed of substrate solution/air and recirculation. Substrate solution: 50 mm ϵ‐caprolactone in 200 mm potassium phosphate buffer pH 8, 5 mm NAD^+^, 100 mm IPA, 1 or 5 mm FAD, 0.1 mm PLP. *P*=atmospheric pressure. Recirculated solution volume 11.37 mL. CalB (Novozym® 435) packed‐bed reactor (CalB PBR): reactor volume 1.09 mL, temperature 37 °C, and residence time 10 min. HLADH‐HeWT‐LpNOX packed‐bed reactor (HLADH‐HeWT‐LpNOX PBR): reactor volume 3.25 mL, temperature 30 °C, and residence time 15 min.

Following the results of the batch biotransformations, the continuous‐flow synthesis was explored with the best substrate, ϵ‐caprolactone. Two continuous flow PBRs were set‐up using, respectively, the immobilized CalB biocatalyst and the co‐immobilized multienzyme system consisting of commercial HLADH, HeWT, and LpNOX, and assembled in tandem to produce 6ACA from ϵ‐caprolactone in continuous flow. A segmented air–liquid flow strategy was implemented to ensure a constant supply of O_2_ to the liquid phase for the LpNOX‐catalyzed oxidation of NADH to yield NAD^+^, as previously reported.^[23]^ A liquid phase containing 50 mm ϵ‐caprolactone, 0.1 mol equiv. NAD^+^, 2 mol equiv. IPA, 0.1 mm PLP, and 1 mm FAD, was flowed into the two serial PBRs with a residence time of 10 min in the first PBR (CalB). An air inlet was placed downstream the first PBR to obtain a segmented 50 : 50 (mL min^−1^ : mL min^−1^) substrate solution/air flow directed into the second PBR (HLADH‐HeWT‐LpNOX) with 15 min residence time (Scheme 2).

**Scheme 3:**
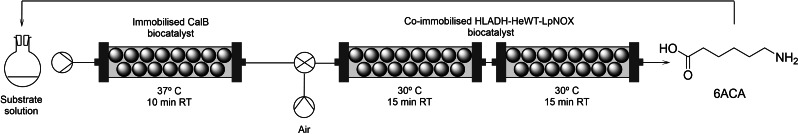
Continuous biocatalytic production of 6ACA from ϵ‐caprolactone in three serial PBRs implementing segmented liquid‐gas flow composed of substrate solution/air and recirculation. Substrate solution: 50 mM ϵ‐caprolactone in 200 mm potassium phosphate buffer pH 8, 5 mm NAD^+^, 100 mm IPA, 1 mm FAD, 0.1 mm PLP. *P*=atmospheric pressure. Recirculated solution volume 11.37 mL. CalB (Novozym® 435) packed‐bed reactor (CalB PBR): reactor volume 1.09 mL, temperature 37 °C, and residence time 10 min. HLADH‐HeWT‐LpNOX packed‐bed reactors (HLADH‐HeWT‐LpNOX PBR): reactor volume 3.25 mL each, temperature 30 °C, and residence time 15 min each.

This biphasic flow allowed the synthesis of 6ACA from ϵ‐caprolactone with only 9 % m.c. Recirculation of the reaction mixture through the two serial PBRs to obtain 4× residence time increased the m.c. to 77 % (Table 7). To improve further the reaction conversion, a third PBR was set‐up using co‐immobilized multienzyme system consisting of commercial HLADH, HeWT, and LpNOX, to increase the residence time without decreasing the flow rate. This PBR was assembled in‐line of the second PBR containing the same biocatalysts (Scheme 3). The addition of a second PBR with HLADH, HeWT, and LpNOX catalysts pushed the m.c. to 26 % after 1 pass through the serial PBRs, however, the m.c. did not increase any further than 77 % after 4 passes (Table 7). We speculate that this might be due the thermodynamic equilibrium reached under these reaction conditions.

**Table 7:** Continuous biocatalytic production of 6ACA from ϵ‐caprolactone implementing segmented liquid‐gas flow composed of substrate solution/air and recirculation.

Serial PBRs assembly	FAD [mm]	Residence time^[a]^	m.c.^[b]^ [%]	STY^[c]^ [g_6ACA_ h^−1^ L^−1^]
Scheme 2	1	1×RT	9	0.90
		2×RT	29	1.44
		4×RT	77	1.92
Scheme 3	1	1×RT	26	1.46
		2×RT	40	1.11
		4×RT	78	1.08
Scheme 2	5	1×RT	34	3.31
		2×RT	48	2.34
		4×RT	74	1.80

[a] RT=residence time. [b] Determined by HPLC by following product formation using a calibration curve. [c] At 50 mm scale. Recirculated solution volume=11.37 mL. CalB (Novozym® 435) packed‐bed reactor (CalB PBR): reactor volume=1.09 mL, *T*=37° C and residence time=10 min. HLADH‐HeWT‐LpNOX packed‐bed reactor (HLADH‐HeWT‐LpNOX PBR): reactor volume=3.25 mL, *T*=30 °C, residence time=15 min.

Next, the concentration of FAD cofactor in the liquid phase was explored, as low concentrations of FAD may result in insufficient availability of FAD if the FAD bound to the enzyme active site is stripped under flow conditions. In fact, in the majority of flavoenzymes, this cofactor is tightly but non‐covalently bound,^[38]^ and poor activity of LpNOX enzyme due to missing FAD has been previously reported,^[39]^ as well as LpNOX activation after incubation with an excess of free FAD.^[39]^ This activation mechanism has also been reported for other NOX enzymes.^[40]^ A substrate solution containing a 5‐fold increased concentration of FAD was then flowed into the two serial PBRs (the first one: CalB with 10 min residence time; the second one: HLADH‐HeWT‐LpNOX with 15 min residence time) with a segmented 50 : 50 substrate solution/air flow directed into the second PBR (Scheme 2). The 5‐times higher FAD concentration increased the m.c. to 34 % after 1 pass, although it did not increase the m.c. after 4 passes (Table 7). Therefore, this cell‐free system consisting of two serial PBRs based on immobilized enzyme biocatalysts (CalB‐HLADH‐HeWT‐LpNOX) yields 3.31 g_6ACA_ h^−1^ L^−1^ STY in continuous flow, without any recirculation, using 50 mm ϵ‐caprolactone as substrate and 0.1 equiv. of both NAD^+^ and FAD cofactors. Remarkably this is the highest yield reported in the (cell‐free) biosynthesis of 6ACA up to date,[^2^, ^3^] and the first example of synthesis of this platform chemical in continuous flow. It is also the first reported route of biosynthesis of 6ACA from ϵ‐caprolactone.

## Conclusion

This work represents one of the first examples of cell‐free biosynthesis of 6‐aminocaproic acid (6ACA), an attractive precursor of ϵ‐caprolactam, which is used to synthesize nylon 6, one of the most common synthetic polymers produced nowadays with an important global market share within the chemical industry. It is also the first multienzyme cascade proposed to synthesize an ω‐amino acid from the corresponding lactone, which is relevant since the synthesis of 4‐, 5‐, and 6‐carbon lactones from lignocellulosic materials has been demonstrated, thus putting steps forwards in the transition from petroleum‐derived chemicals to renewable feedstocks in the manufacturing of polymer building blocks. Furthermore, this serial packed‐bed reactor (PBR) system based on immobilized enzyme biocatalysts provides the highest yield in the (cell‐free) biosynthesis of 6ACA up to date,[^2^, ^3^] and constitutes the first biosynthetic strategy to fabricate 6ACA in continuous flow, which further promotes the integration of flow technologies within the chemical industry, with potential application in the synthesis of nylon precursors. An in‐line purification strategy to separate the product from the main stream would be a valuable addition to this system.^[41]^ Hence, the method described here has significant potential in the synthesis of this platform chemical and in the incorporation of greener synthetic approaches in the polymer industry.^[10]^ Ongoing research is focusing on the assembly of an extended enzymatic cascade which will enable the synthesis of a range of ω‐amino acids starting from the diols^[22]^ instead of the lactones, and replacing *Candida antarctica* lipase B, which has reduced activity with the smaller lactones, with the highly active lactonases, which will expand the range of valuable products accessible via this strategy.

## Experimental Section

### Materials

All chemicals and reagents used for this work were purchased from Sigma‐Aldrich unless specified otherwise. *E. coli* BL21(DE3) Star competent cells were purchased from Themo Fisher Scientific and propagated in house. Anydrotetracycline was purchased from Cayman Chemical Company. StrepTrap HP 5 mL and HisTrap HP 1 mL columns were acquired from GE Healthcare. Nicotinamide adenine dinucleotide oxidized (NAD^+^) and reduced (NADH) forms were purchased from Apollo Scientific. Relisorb® EP400SS was donated by Resindion. Polyethyleneimine (PEI) (*M*_n_=60000, 50 % aq. Solution, branched) was purchased from Acros Organics. 6‐Hydroxycaproic acid was purchased from Acros Organics. 5‐Aminovaleric acid and 4‐aminobutanoic acid were acquired from Apollo Scientific. 6‐Aminocaproic acid, ϵ‐caprolactone acid, and γ‐butyrolactone were purchased from Sigma. δ‐Valerolactone was acquired from Alfa Aesar. Commercial horse liver alcohol dehydrogenase was purchased from Sigma. Commercial immobilized CalB preparation (Novozym® 435) was acquired from Novozymes.

### Enzyme expression and purification

The plasmid harboring HLADH (pASK‐IBA5plus‐HLADH),^[23]^ HeWT,^[31]^ and LpNOX^[39]^ were transformed into *E. coli* BL21(DE3) Star. 1 Enzyme expression and purification was developed as previously described.^[23]^

### Enzymatic activity assays and determination of protein concentration

All enzyme assays were performed in triplicate in 96‐well microplates (unless specified otherwise) using Epoch 2 Microplate Spectrophotometer (Biotek). For HLADH, HeWT, and LpNOX, the activity assays were performed as previously indicated,[^23^, ^31^, ^33^] at 25 °C and pH 8 (50 mm potassium phosphate buffer). HLADH and HEWT protein concentration were determined by UV absorption at 280 nm using the Epoch Take3 Micro‐Volume Plate. The extinction coefficients 22460 and 62340 m ^−1^ cm^−1^, at 280 nm, measured in water, were respectively estimated for HLADH and HEWT by ExPASy ProtParam tool, accessible from the ExPASy website (www.expasy.ch). The LpNOX concentration was determined using a Bradford assay with bovine serum albumin (BSA) as standard, as previously reported.^[39]^

### Sequential functionalization of methacrylate carrier and co‐immobilization of HLADH, HEWT, and LpNOX

3 g of Relisorb® EP400SS was functionalized with glyoxyl groups as previously reported.^[23]^ Resulting glyoxyl groups were quantified following the described protocol.^[42]^

A 30 mL solution containing 45 mg of pure HLADH, or 40 mg of commercial HLADH, in 100 mm NaHCO_3_ pH 10 was added to the resin containing glyoxyl groups (3 g). The suspension was incubated for 1 h at 4 °C under mild agitation, and 30 mg of NaBH_4_ was then added and further incubated for 30 min at 4 °C under mild agitation. HLADH activity in the remaining liquid phase before NaBH_4_ addition was measured. The immobilized HLADH biocatalyst was filtered and washed with H_2_O. The specific activity of the immobilized HLADH enzyme was determined as described in the Supporting Information (Tables S1 and S2). The rest was added to a 30 mL solution of 300 mm ethylenediamine (EDA) in 100 mm NaHCO_3_ pH 8.5. The suspension was incubated for 2 h at room temperature under mild agitation. The EDA‐modified immobilized HLADH biocatalyst was filtered and washed with H_2_O. A 30 mL solution containing 22.5 mg (or 18 mg when using commercial HLADH) of pure HEWT in 50 mm potassium phosphate buffer 0.1 mm PLP pH 8 was added to this biocatalyst. After 4 h incubation at 4 °C under mild agitation, the resulting co‐immobilized HLADH‐HEWT biocatalyst was filtered, washed with H_2_O, and added to a 60 mL solution of 0.05 g mL^−1^ PEI in 25 mm potassium phosphate buffer pH 7.5. The suspension was incubated for 16 h at room temperature under mild agitation, and the resulting co‐immobilized HLADH‐HEWT biocatalyst was filtered and washed with H_2_O. The specific HLADH and HeWT activities of the co‐immobilized HLADH‐HEWT biocatalyst were determined as described in the Supporting Information (Tables S1 and S2). A 30 mL solution containing 15 mg of pure LpNOX in 25 mm potassium phosphate buffer 0.1 mm FAD pH 7 was added to this biocatalyst. After 4 h incubation at 4 °C under mild agitation, the resulting co‐immobilized HLADH‐HEWT‐LpNOX biocatalyst was filtered and washed with H_2_O. The specific LpNOX activity of the co‐immobilized LpNOX‐HEWT‐HLADH biocatalyst was determined as described in Supporting Information (Tables S1 and S2).

### Batch reactions of synthesis of 6ACA from 6‐hydroxycaproic acid

Batch reactions with pure soluble enzymes were performed at 30 °C in 1 mL of reaction mixture containing 10, 50, or 100 mm 6‐hydroxycaproic acid, 0.1 equiv. NAD^+^ (1, 5, or 10 mm), 2 equiv. IPA (20, 100, or 200 mm), 0.1 mm PLP, 1 mm FAD, 50 mm potassium phosphate buffer pH 8, HLADH (1.5 mg mL^−1^), HEWT (0.75 mg mL^−1^), and LpNOX (0.25 mg mL^−1^). Batch reactions with the co‐immobilized multienzyme system were performed at 30 °C in 1 mL of reaction mixture containing 10, 50, or 100 mm 6‐hydroxycaproic acid, 0.1 equiv. NAD^+^ (1, 5, or 10 mm), 2 equiv. IPA (20, 100, or 200 mm), 0.1 mm PLP, 1 mm FAD, 50 mm potassium phosphate buffer pH 8, and 0.1 g of the co‐immobilized HLADH‐HEWT‐LpNOX biocatalyst. The reactions were monitored by HPLC, as described in the “Analytical methods” section.

### Batch reactions of hydrolysis of lactones

Batch reactions with the commercial immobilized CalB preparation were performed at 37 °C in 0.4 mL of reaction mixture containing 100 mm ϵ‐caprolactone, δ‐valerolactone, or γ‐butyrolactone, 100 mm potassium phosphate buffer pH 8, and 10 mg of immobilized CalB biocatalyst. The reactions were monitored by ^1^H NMR spectroscopy^[37]^ (without pre‐saturation of the water peak) after addition of 200–300 μL of D_2_O to the sample and filtration through a cotton plug. See Figures S6–S8 for representative ^1^H NMR spectra.

### Continuous‐flow reactions of hydrolysis of lactones

An Omnifit glass column (6.6 mm i.d. ×150 mm length) was filled with the immobilized CalB biocatalyst to set up a PBR (CalB PBR; reactor volume=2.5 mL). Temperature was set to 37 or 45 °C. A 100 mm substrate solution (ϵ‐caprolactone, δ‐valerolactone, or γ‐butyrolactone) in 50 mm potassium phosphate buffer pH 8.0 and 4 % DMSO was directed into the CalB PBR. The flow rate was varied to allow a residence time of 10–20 min. The reactions were monitored by ^1^H NMR spectroscopy^[37]^ (without pre‐saturation of the water peak), after addition of 280 μL of D_2_O to 400 μL of flow‐through.

### Batch reactions of synthesis of 6ACA, 5AVA, and GABA from lactones

Batch reactions with immobilized biocatalysts were performed at 30 °C in 1 mL of reaction mixture containing 50 mm ϵ‐caprolactone, δ‐valerolactone, or γ‐butyrolactone, 0.1 equiv. NAD^+^ (5 mm), 2 equiv. IPA (100 mm), 0.1 mm PLP, 1 mm FAD, 50 or 200 mm potassium phosphate buffer pH 8, 0.01 g of the immobilized CalB biocatalyst, and 0.1 g of the co‐immobilized HLADH‐HEWT‐LpNOX biocatalyst. The reactions were monitored by HPLC, as described in the “Analytical methods” section.

### Continuous‐flow synthesis of 6ACA from ϵ‐caprolactone

An Omnifit glass column (6.6 mm i.d. ×150 mm length) was filled with the immobilized CalB biocatalyst to set up a PBR (CalB PBR; reactor volume=1.09 mL). Another Omnifit glass column (6.6 mm i.d. ×150 mm length) was filled with approximately 3 g the co‐immobilized HLADH‐HEWT‐LpNOX biocatalyst to set up a second PBR (HLADH‐HEWT‐LpNOX PBR; reactor volume=3.25 mL). In all the reactions performed, temperature was set to 37 °C for CalB PBR and to 30 °C for HLADH‐HEWT‐LpNOX PBR. A 50 mm substrate solution (ϵ‐caprolactone) containing 5 mm NAD^+^, 100 mm IPA, 0.1 mm PLP, 1 or 5 mm FAD in 200 mm potassium phosphate buffer pH 8.0 was prepared. For the flow reactions with 2 serial PBRs, this substrate solution was directed into the CalB PBR. The flow rate was 108 μL min^−1^ to allow a residence time of 10 min in CalB PBR. Air was flowed into the second PBR (HLADH‐HeWT‐LpNOX PBR) at the same flow rate with a second peristaltic pump and mixed to the exiting flow stream of CalB PBR using a T‐junction. The resulting 50 : 50 substrate solution/air segmented flow was directed into the second PBR (HLADH‐HeWT‐LpNOX PBR) to allow a residence time of 15 min. For the flow reactions with 3 serial PBRs, a third PBR containing co‐immobilized HLADH‐HEWT‐LpNOX biocatalyst was set up (HLADH‐HEWT‐LpNOX PBR; reactor volume=3.25 mL) and placed in‐line with the second PBR. The exiting flow stream of the second or the third PBR was placed in the same container as the substrate solution to allow recirculation. Samples were taken from this container and analyzed by HPLC, as described in the “Analytical methods” section. After one pass of the 11.37 mL substrate solution (after 105 min), a sample was taken and analyzed by HPLC. Other samples were taken after 2 and 4 passes (after 3.5 and 7 h, respectively).

### Analytical methods

Batch and flow reactions of the synthesis of ω‐amino acids (6ACA, 5AVA, and GABA) were monitored by HPLC following a fluorenylmethyloxycarbonyl (FMOC) derivatization protocol. 100 μL sample with a maximum concentration of ω‐amino acid of 10 mm were added to 200 μL 100 mm borate buffer pH 9 followed by the addition of 400 μL 15 mm FMOC−Cl in acetonitrile. The reaction was vortexed for 30 s and then 200 μL of the reaction mixture were added to 400 μL MilliQ water and 400 μL acetonitrile. The samples were analyzed by HPLC (ThermoFisher Ultimate 3000 Reverse‐phase HPLC with diode array detector) on a Waters XBridge C18 column (3.5 μm, 2.1×150 mm), measuring at 210 nm, using a gradient method from 40 : 95 (H_2_O/MeCN 0.1 % TFA) over 4 min with a flow rate of 0.8 mL min^−1^. The retention times of the different compounds were: 6ACA 2.26 min and 5AVA 1.73 min. For GABA, a gradient method from 30 : 45 (H_2_O/MeCN 0.1 % TFA) over 4 min with a flow rate of 0.8 mL min^−1^ was used. The retention time was 4.17 min. Molar conversions were calculated through a standard curve of the products.

## Conflict of interest

The authors declare no conflict of interest.

1

## Supporting information

As a service to our authors and readers, this journal provides supporting information supplied by the authors. Such materials are peer reviewed and may be re‐organized for online delivery, but are not copy‐edited or typeset. Technical support issues arising from supporting information (other than missing files) should be addressed to the authors.

Supporting Information

## Data Availability

The data that support the findings of this study are available from the corresponding author upon reasonable request.
