# Supplementary material for: Biocatalytic Production of a Nylon 6 Precursor from Caprolactone in Continuous Flow
Source: ChemSusChem. 2022 Jun 28;15(16):e202200811. doi: 10.1002/cssc.202200811 (PMC9546309; doi:10.1002/cssc.202200811)
Supplement: Supplementary file 1 — Supporting Information [file CSSC-15-0-s001.pdf]

ChemSusChem

Supporting Information

Biocatalytic Production of a Nylon 6 Precursor from Caprolactone in Continuous Flow

Maria Romero-Fernandez, Christian M. Heckmann, and Francesca Paradisi*© 2022 The Authors. ChemSusChem published by Wiley-VCH GmbH. This is an open access article under the terms of the Creative Commons Attribution License, which permits use, distribution and reproduction in any medium, provided the original work is properly cited.

Batch reactions of synthesis of 6-aminocaproic acid (6ACA) from 6-hydroxycaproic acid with different biocatalysts

Batch reactions with pure soluble enzymes were performed at 30º C in 5 mL of reaction mixture containing 10 mM 6-hydroxycaproic acid in 50 mM potassium phosphate buffer pH 8, 1 mM NAD^+, 20 mM IPA, 0.1 mM PLP, 1 mM FAD, alcohol oxidase from *Pichia pastoris,* alcohol oxidase from *Candida boidinii*, or glucose oxidase from *Aspergilus niger* (0.7 mg mL^{-1}), and HEWT (0.9 mg mL^{-1}). The reactions were monitored by HPLC following a FMOC-Cl derivatisation protocol. 100 μL sample were added to 200 μL 100 mM borate buffer pH 9 followed by the addition of 400 μL 15 mM FMOC in acetonitrile. The reaction was vortexed for 30 seconds to proceed and then 200 μL of the reaction mixture were added to 400 μL MilliQ water and 400 μL acetonitrile. The samples were analysed on a Waters XBridge C18 column (3.5μm, 2.1 × 150mm), measuring at 210 nm, using a gradient method from 40 : 95 (H_2O : MeCN 0.1%TFA) over 4 minutes with a flow rate of 0.8 mL min^{-1}. The retention time of 6ACA was 2.26 min. Molar conversions were calculated through a standard curve of the product.

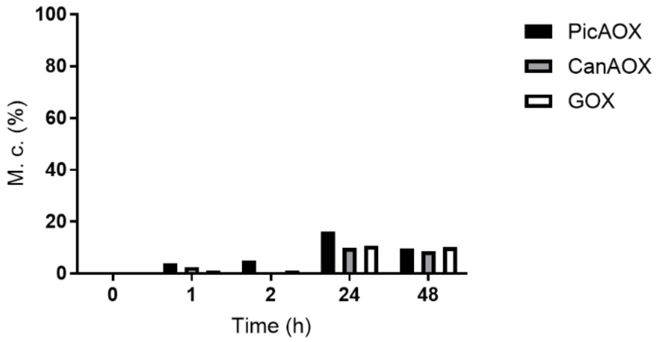

Figure S1. Synthesis of 6ACA in batch reactions catalysed by soluble AOX from *Pichia pastoris,* AOX from *Candida boidinii*, or glucose oxidase from *Aspergilus niger* (0.7 mg mL^{-1}), and HEWT (0.9 mg mL^{-1}). Reaction conditions: 10 mM, 6-hydroxycaproic acid in 50 mM potassium phosphate buffer pH 8, 0.1 equivalents NAD^+ (1 mM), 2 equivalents IPA (20 mM), 1 mM FAD, and 0.1 mM PLP. T= 30 °C. Reaction volume = 5 mL. Mean values of triplicate reactions.

Co-immobilisation of HLADH, HeWT and LpNOX

The sequential co-immobilisation of purified HLADH (15 mg $g_{carrier}^{-1}$), HeWT (7.5 mg $g_{carrier}^{-1}$) and LpNOX (5 mg $g_{carrier}^{-1}$) was developed as described in *Sequential functionalization of methacrylate carrier and co-immobilisation of HLADH, HEWT and LpNOX* section. Likewise, the sequential co-immobilisation of commercial HLADH (20 – 40 mg $g_{carrier}^{-1}$), HeWT (3 – 5 mg $g_{carrier}^{-1}$) and LpNOX (5 mg $g_{carrier}^{-1}$) was developed following the same protocol.

The specific immobilised enzyme activity was measured using the same conditions as for the free enzyme, as previously reported.[1] For HLADH and HEWT, 20 mg of the immobilised biocatalyst were added to a 10 mL reaction mixture. Every 2 min during 10 min, a sample was taken to measure the absorbance at 340 nm or 245 nm, for HLADH or HEWT, respectively. For LpNOX, 20 mg of the immobilised biocatalyst were added to 40 mL of reaction mixture, and the same protocol was followed. The specific activity of HLADH, HEWT and LpNOX immobilised biocatalysts (U $g_{carrier}^{-1}$) was defined as: the formation of NADH in μmol per minute and g of biocatalyst (HLADH); the formation of acetophenone in μmol per minute and g of biocatalyst (HEWT); and the depletion of NADH in μmol per minute and g of biocatalyst (LpNOX).

The immobilised activity (U $g_{carrier}^{-1}$) was calculated as the difference between initial offered activity per gram of carrier and remaining activity in the liquid phase per gram of carrier at the immobilisation end point. The immobilisation yield (%) was calculated as the ratio between immobilised activity and initial offered activity per gram of carrier. The recovered activity (%) was calculated as the ratio between the specific activity of the immobilised enzyme and the immobilised activity.

Table S1. Immobilisation of 15 mg purified HLADH, 7.5 mg HEWT and 5 mg LpNOX per gram of functionalized porous bead carrier based on a polymethacrylate matrix.

	Immobilisation yield (%)	Recovered activity (%)	Specific immobilised activity (U $g_{carrier}^{-1}$)
HLADH	95	11	1.74
HeWT	86	26	4.16
LpNOX	74	34	13.12

Table S2. Immobilisation of 20 - 40 mg commercial HLADH, 3 - 6 mg HEWT and 5 mg LpNOX per gram of functionalized porous bead carrier based on a polymethacrylate matrix.

Loading commercial HLADH (mg $g_{carrier}^{-1}$)	Loading HeWT (mg $g_{carrier}^{-1}$)	Enzymes	Immobilisation yield (%)	Recovered activity (%)	Specific immobilised activity (U $g_{carrier}^{-1}$)
20	3	HLADH	100	73	0.97
		HeWT	76	37	2.38
		LpNOX	63	46	32.87
20	6	HLADH	100	82	1.09
		HeWT	78	26	3.04
		LpNOX	64	47	34.35
40	6	HLADH	100	75	2.00
		HeWT	81	44	5.95
		LpNOX	86	70	49.19

Optimisation of the co-immobilised multienzyme system based on commercial HLADH, HeWT and LpNOX for the synthesis of 6ACA from 6-hydroxycaproic acid

a) Ratio of commercial HLADH to HeWT loadings

The sequential co-immobilisation of commercial HLADH (20 mg $g_{carrier}^{-1}$), HeWT (3 – 6 mg $g_{carrier}^{-1}$) and LpNOX (5 mg $g_{carrier}^{-1}$) was developed as described in *Sequential functionalization of methacrylate carrier and co-immobilisation of HLADH, HEWT and LpNOX* section. Two loadings of HeWT were tested, 3 mg $g_{carrier}^{-1}$ and 6 mg $g_{carrier}^{-1}$. The immobilization yield, recovered activity, and specific immobilised activity for immobilised HLADH, HeWT and LpNOX were calculated (Table S2). The resulting co-immobilised biocatalysts were used to catalyse the synthesis of 6ACA from 6-hydroxycaproic acid in batch reactions at 30 ºC in 1 mL reaction mixture containing 10 mM 6-hydroxycaproic acid in 50 mM potassium phosphate buffer pH 8, 1 mM NAD^+, 20 mM IPA, 0.1 mM PLP, 1 mM FAD, and 0.1 g of the resulting co-immobilised biocatalysts. The reactions were monitored by HPLC following a FMOC-Cl derivatisation protocol. 100 µL sample were added to 200 µL 100 mM borate buffer pH 9 followed by the addition of 400 µL 15 mM FMOC in acetonitrile. The reaction was vortexed for 30 seconds to proceed and then 200 µL of the reaction mixture were added to 400 µL MilliQ water and 400 µL acetonitrile. The samples were analysed on a Waters XBridge C18 column (3.5µm, 2.1 × 150mm), measuring at 210 nm, using a gradient method from 40 : 95 (H_2O : MeCN 0.1%TFA) over 4 minutes with a flow rate of 0.8 mL min^{-1}. The retention time of 6ACA was 2.26 min. Molar conversions were calculated through a standard curve of the product.

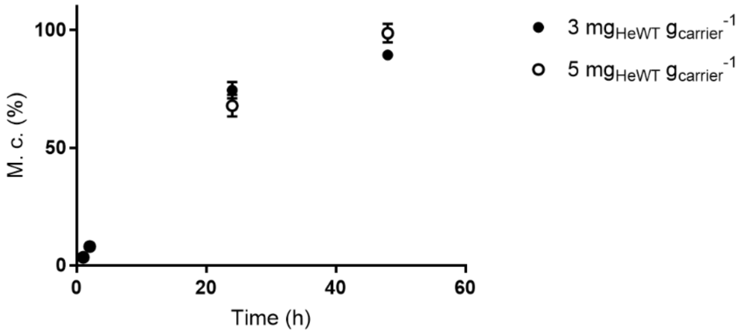

Figure S2. Synthesis of 6ACA in batch reaction catalysed by 0.1 g of the co-immobilised multienzyme system consisting of commercial HLADH, HEWT and LpNOX per mL of reaction. Reaction conditions: 10 mM 6-hydroxycaproic acid in 50 mM potassium phosphate buffer pH 8, 0.1 equivalents NAD^+ (1 mM), 2 equivalents IPA (20 mM), 1 mM FAD, and 0.1 mM PLP. T= 30 °C. Reaction volume = 1 mL. Mean values of triplicate reactions.

b) Loading of commercial HLADH

The sequential co-immobilisation of commercial HLADH (20 – 40 mg $g_{carrier}^{-1}$), HeWT (3 – 6 mg $g_{carrier}^{-1}$) and LpNOX (5 mg $g_{carrier}^{-1}$) was developed as described in *Sequential functionalization of methacrylate carrier and co-immobilisation of HLADH, HEWT and LpNOX* section. Three loadings of commercial HLADH were tested, 20 mg $g_{carrier}^{-1}$, 30 mg $g_{carrier}^{-1}$, and 40 mg $g_{carrier}^{-1}$. To keep the selected commercial HLADH to HeWT ratio (20 mg commercial HLADH to 3 mg HeWT), HeWT loading was varied: 3 mg $g_{carrier}^{-1}$, 4.5 mg $g_{carrier}^{-1}$, and 6 mg $g_{carrier}$-1, respectively. A sample of the liquid phase at start and at the end of the immobilisation of HLADH was taken and protein concentration was measured by Bradford (Table S3). Total protein content of the liquid phase at start and at the end of immobilisation was analysed by SDS-page electrophoresis. A sample of the resulting immobilised biocatalyst was resuspended in MilliQ water (keeping the same ratio of liquid to resin as in the immobilisation) and was incubated for 30 min at 90 ºC. A sample of the liquid phase was taken, and protein content was analysed by SDS-page electrophoresis (Figure S4).

Table S3. Protein concentration in the liquid phase at the start and the end of the immobilisation of the commercial HLADH preparation.

Loading commercial HLADH (mg $g_{carrier}^{-1}$)	Protein concentration at the start of immobilisation (%)	Protein concentration at the end of immobilisation (%)
20	100	22
30	100	27
40	100	25

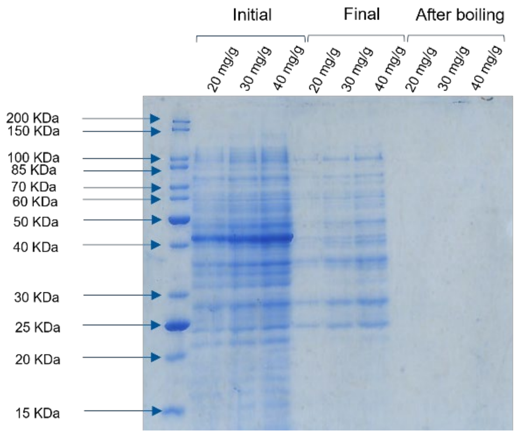

Figure S3. SDS-page electrophoresis of samples taken from the liquid phase at the start and at the end of HLADH immobilisation (with different loadings), and from the liquid phase after boiling the resulting immobilised biocatalyst in the same volume of buffer.

With the highest loading obtained for the commercial HLADH preparation, 40 mg $g_{carrier}^{-1}$, a HeWT loading 6 mg $g_{carrier}^{-1}$ was used to keep the selected ratio of commercial HLADH to HeWT (20 mg commercial HLADH to 3 mg HeWT, Figure S3). The immobilization yield, recovered activity, and specific immobilised activity for immobilised HLADH, HeWT and LpNOX were calculated (Table S2). The resulting co-immobilised biocatalyst was used to catalyse the synthesis of 6ACA from 6-hydroxycaproic acid in batch reactions at 30 ºC in 1 mL reaction mixture containing 10 mM 6-hydroxycaproic acid in 50 mM potassium phosphate buffer pH 8, 1 mM NAD^+, 20 mM IPA, 0.1 mM PLP, 1 mM FAD, and 0.1 g of the resulting co-immobilised biocatalyst. The reactions were monitored by HPLC following a FMOC-Cl derivatisation protocol. 100 µL sample were added to 200 µL 100 mM borate buffer pH 9 followed by the addition of 400 µL 15 mM FMOC in acetonitrile. The reaction was vortexed for 30 seconds to proceed and then 200 µL of the reaction mixture were added to 400 µL MilliQ water and 400 µL acetonitrile. The samples were analysed on a Waters XBridge C18 column (3.5µm, 2.1 × 150mm), measuring at 210 nm, using a gradient method from 40 : 95 (H_2O : MeCN 0.1%TFA) over 4 minutes with a flow rate of 0.8 mL min^{-1}. The retention time of 6ACA was 2.26 min. Molar conversions were calculated through a standard curve of the product.

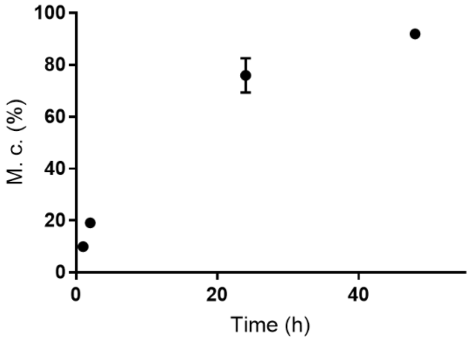

Figure S4. Synthesis of 6ACA in batch reaction catalysed by 0.1 g of the co-immobilised multienzyme system consisting of commercial HLADH, HEWT and LpNOX per mL of reaction. Reaction conditions: 10 mM 6-hydroxycaproic acid in 50 mM potassium phosphate buffer pH 8, 0.1 equivalents NAD^+ (1 mM), 2 equivalents IPA (20 mM), 1 mM FAD, and 0.1 mM PLP. T= 30 °C. Reaction volume = 1 mL. Mean values of triplicate reactions.

Calculation of space time yield and catalyst productivity

The space time yield (STY) in continuous flow reactions was calculated as follows:

$$STY\ (g_{6ACA}\ L^{-1}\ h^{-1}) = \frac{Substrate\ concentration_{inlet}\ (M)\ \times\ Conversion_{outlet}\ (\%) \times\ Flow\ rate\ (L\ h^{-1}) \times MW_{6ACA}\ (g\ mol^{-1})}{Reactor\ volume\ (L)\ \times\ 100\ \times Number\ of\ passes}$$

The flow rate was 0.0069 L h-1 for the reaction with 2 serial PBRs and 1 mM FAD; and 0.00648 L h^{-1} for the reactions with 3 serial PBRs and 1 mM FAD; and 2 serial PBRs and 5 mM FAD. The CalB reactor volume is 0.00109 L for the three reactions, and the HLADH-HeWT-LpNOX reactor volume is 0.003455 L for the reaction with 2 serial PBRs and 1 mM FAD, and 0.00325 L for the reactions of 3 serial PBRs and 1 mM FAD and 2 serial PBRs and 5 mM FAD. Molecular weight of 6ACA is 131.17 g mol^{-1}.

The STY in batch reactions was calculated as follows:

$$STY\ (g_{6ACA}\ L^{-1}\ h^{-1}) = \frac{Substrate\ concentration\ (M)\ \times\ Conversion\ (\%) \times MW_{6ACA}\ (g\ mol^{-1})}{Reaction\ time\ (h)\ \times\ 100}$$

The catalyst productivity in batch reactions at a reaction time point was calculated as follows:

$$Catalyst\ productivity\ \left(\mu mol_{6ACA}\ h^{-1}\ mg_{enzyme}{}^{-1}\right) = \frac{Reaction\ Volume\ (L)\ \times\ Substrate\ concentration\ (M)\ \times\ Conversion\ (\%) \times\ 1{,}000{,}000}{Time\ (h) \times\ Total\ enzyme\ (mg) \times 100}$$

In the batch reactions of synthesis of 6ACA (1 mL reaction volume) catalysed by 0.1 g of the co-immobilised multienzyme system consisting of commercial HLADH (loading 40 mg $g_{carrier}^{-1}$), HEWT (loading 6 mg $g_{carrier}^{-1}$) and LpNOX (loading 5 mg $g_{carrier}^{-1}$) per mL of reaction, the Total enzyme is 5.1 mg.

Representative ^{1}H-NMR spectra of hydrolysis reactions of the lactones

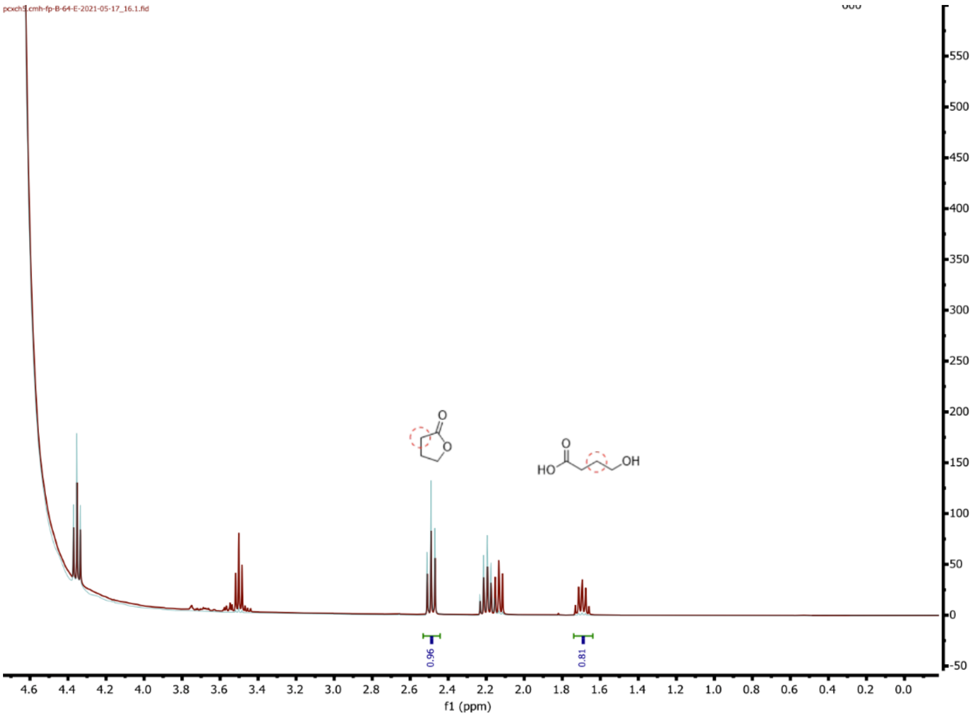

Figure S6. ^{1}H-NMR spectra of a reaction mixture of γ-butyrolactone with (brown) and without (blue) Cal-B.

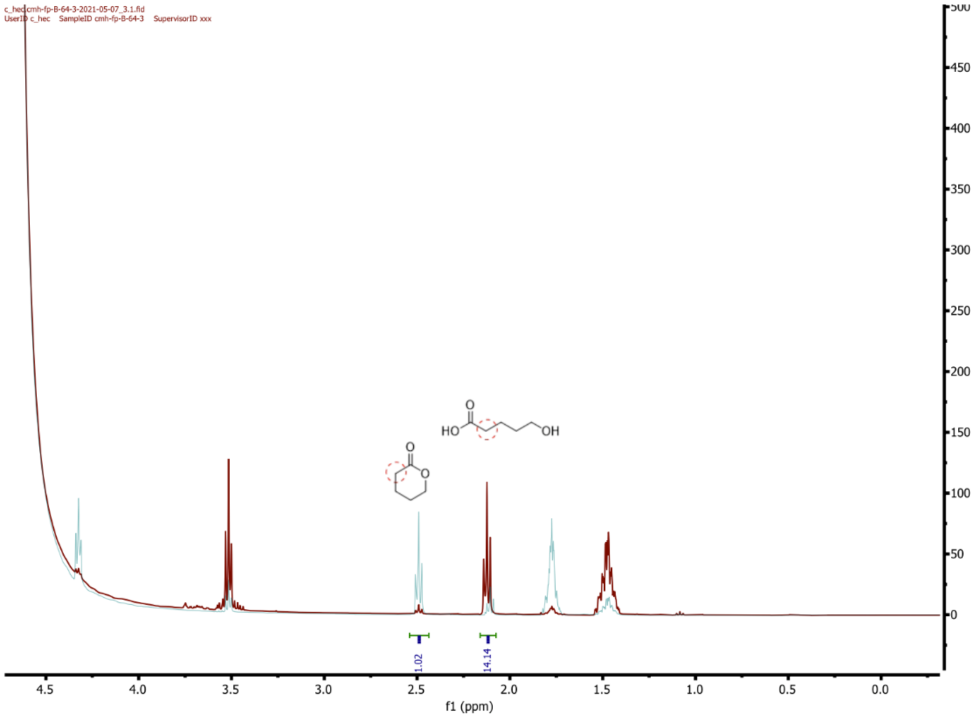

Figure S7. ^{1}H-NMR spectra of a reaction mixture of δ-valerolactone with (brown) and without (blue) Cal-B.

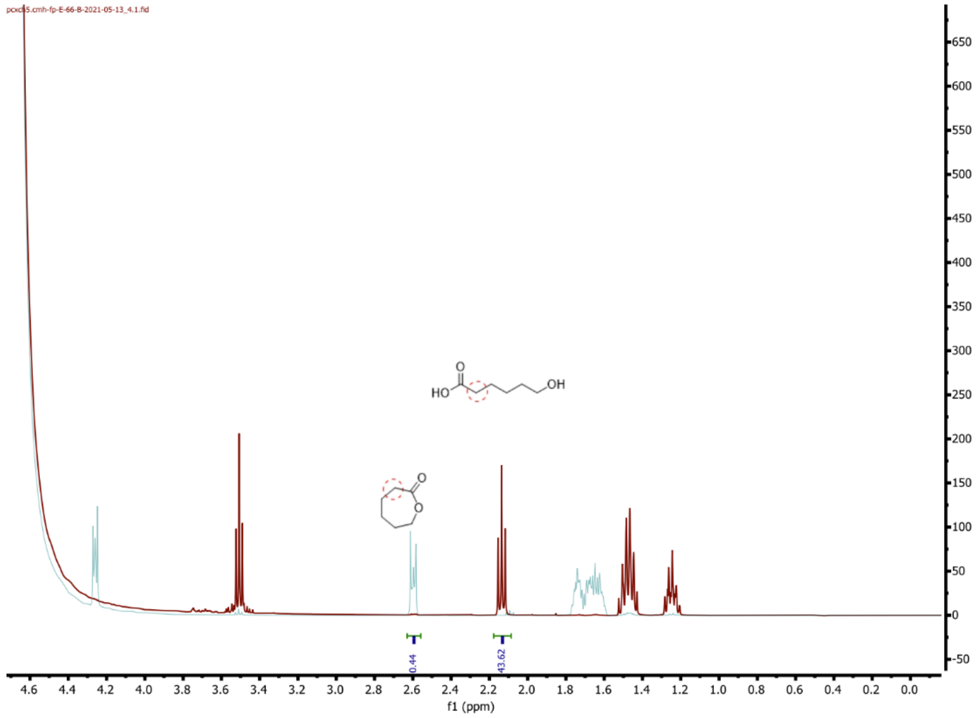

Figure S8. ^{1}H-NMR spectra of a reaction mixture of ε-caprolactone with (brown) and without (blue) Cal-B.

Supporting references

[1] M. Romero-Fernandez, F. Paradisi, *Green Chem.* **2021**, *23*, 4594–4603.
